# Thermal diffusivity modulation driven by the interfacial elastic dynamics between cellulose nanofibers†

**DOI:** 10.1039/c9na00734b

**Published:** 2020-01-20

**Authors:** Kojiro Uetani, Shogo Izakura, Hirotaka Koga, Masaya Nogi

**Affiliations:** The Institute of Scientific and Industrial Research (ISIR), Osaka University Mihogaoka 8-1, Ibaraki-shi Osaka 567-0047 Japan uetani@eco.sanken.osaka-u.ac.jp; Graduate School of Engineering, Osaka University Mihogaoka 8-1, Ibaraki-shi Osaka 567-0047 Japan

## Abstract

Thermal transport modulating materials show great potential to address the heat problems in a wide range of engineering fields. However, tuning the thermal conductivity of solid-state materials is practically difficult because it requires specific or extreme stimulation, such as chemical composition change, a phase transition, or large applied fluctuations, to change the internal bulk structures. Here, we report reversible switching of the in-plane thermal diffusivity of densely packed cellulose nanofiber (CNF) films by ∼15% by simple mechanical strain as small as 0.3%. From analysis of the stress relaxation profiles and the different bulk densities of the CNF films, the interfacial elastic dynamics between the strongly hydrogen bonded CNFs were found to exhibit thermal diffusivity modulation by tuning the interfacial thermal resistance, rather than changing the bulk structure of the CNFs. Our concept of interfacial-elasticity-driven thermal diffusivity switching has the potential to enhance the on/off rate and extensibility toward practical use owing to the high designability of the interfacial conditions.

## Abstract 2

This study reports that the nanoscale interfacial elastodynamics between cellulose nanofibers dynamically modulate the macroscopic thermal diffusivity.

## Graphical abstract

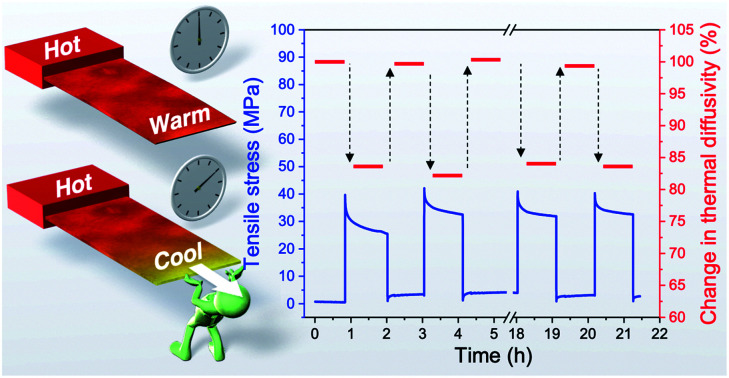

Thermal transport modulators accelerate various thermal techniques in a wide range of fields, including phonon engineering, thermal management, and energy harvesting. In particular, macroscopic solid-state materials that show dynamic thermal conductivity switching performance in response to small and simple external stimulation are of great importance for key thermal techniques to address the heat problems regarding exhaustion, recovery, and reuse of heat. Several studies have reported materials whose thermal conductivities respond to external stimuli. However, some of these materials require specific and extreme stimuli, including large displacements,^1^ hydration,^2^ lithiation,^3^ a magnetic field,^4^ and large temperature changes,^5,6^ and others are nanoscale materials, such as polyethylene nanofibers,^5^ ferroelectric thin films,^7^ silicon nanowires,^8^ and silicon nitride thin films.^9^ Because of the difficulties in changing the internal structures of bulk solid-state materials for the thermal conductivity to respond, it is a major challenge to realize macroscopic thermal conductivity modulating materials based on unconventional mechanisms in response to small and simple stimulations.

Phonons and electrons are clearly different as heat transport media, but sometimes the analogy between the heat transport and electric current becomes a suitable viewpoint. Harada *et al.*^10^ reported a strain sensor that exhibits an electric resistance change derived from the change in the interfacial distance between the silver nanoparticle conductive filler connected by carbon nanotubes in the polymer matrix in response to mechanical displacement. Kapitza resistance occurs in macroscopic phononic thermally conductive materials owing to phonon scattering at the interior interfaces, such as grain boundaries^11,12^ or dissimilar interfaces,^13^ and it greatly affects the apparent thermal conductivity. To design macroscopic thermal transport switching materials triggered by simple and small stimuli, the mechanical stimuli need to cause reversible elastic deformation at the interior interfaces to express clear responses in the thermal conduction properties, rather than at the bulk parts of the solids, which are often the target for various materials.^8,9,14–22^

Herein, we investigated *nata-de-coco*-derived bacterial cellulose nanofiber (CNF) films as macroscopic thermally conductive materials with a large number of interfaces between the fibers. Biologically produced CNFs consisting of cellulose-I-type extended chain crystals with thicknesses of 3 to 20 nm, which cannot be artificially produced, have few defects according to high-resolution micrographs of the crystal lattice.^23^ This highly sophisticated regularity at the molecular level results in ∼10 times anisotropy of the thermal conductivity between the long and short axes,^24,25^ and their films exhibit smoother heat transfer with 3–10 times higher thermal conductivities than conventional polymeric materials.^26–28^

A noteworthy advantage of CNF films is that they have high mechanical toughness despite containing a very large number of interfaces between the crystalline fibers. The densely packed CNF films have high Young's modulus of more than 10 GPa and high strength of ∼200 MPa,^29,30^ and they can also maintain constant modulus and yield stress at each cycle of repetitive stress loading–unloading experiments^31,32^ to exhibit extremely high toughness.^33^ Because the modulus and strength of the constitutive CNFs are 140–150 GPa (ref. 34) and 2–6 GPa,^35^ respectively, the toughness of CNF films is thought to be largely exerted by the interfaces between the CNFs. This interfacial toughness is greatly superior to that of usual engineering materials, because materials containing numerous interfaces between the filler and matrix or grain boundaries easily become very weak and fragile.^36,37^ The CNFs are strongly bound together by hydrogen bonds to form films and simultaneously induce phonon scattering to cause theoretical interfacial thermal resistance of 9.4–12.6 m^2^ K GW^−1^, which is about one-eighth that of carbon nanotubes.^24^ Taking into account both the toughness and low interfacial thermal resistance, application of a simple mechanical force could elastically regulate the thermal resistance along with the interfacial bonding forces to modulate the thermal conduction performance. Consequently, we found that CNF films reversibly switch the in-plane thermal diffusivity by ∼15% in response to a small strain of ∼0.3% at tensile stress of 30–40 MPa. Realization of dynamic thermal transport modulators derived from the interfacial elastic dynamics could assist in development and implementation of key thermal techniques.

The principal technology of our approach is an integrated system to measure the thermal diffusivity of macroscopic bulk materials under applied mechanical stress. The concept is shown in Fig. 1a. The film sample is set on a jig to apply stress. While maintaining the strain, the thermal diffusivities in the *x*, *y*, and *z* directions are independently measured by non-contact laser spot periodic heating radiation thermometry (TA33 thermowave analyzer, Bethel Co., Ltd., Ibaraki, Japan). The specially designed jig (TJ-161, Bethel Co., Ltd.) allows application of tensile stress to the sample by manually rotating the handle, and the strain remains constant after releasing the handle (Fig. 1b). This jig was designed to place the sample at the focusing level of both the heating laser and radiation thermometry detection system during stress application. We carefully determined the mounting position of the jig on the thermowave analyzer without touching the sample chucks on the stage or the laser filter cylinder on the jig (Fig. 1c).

**Fig. 1:**
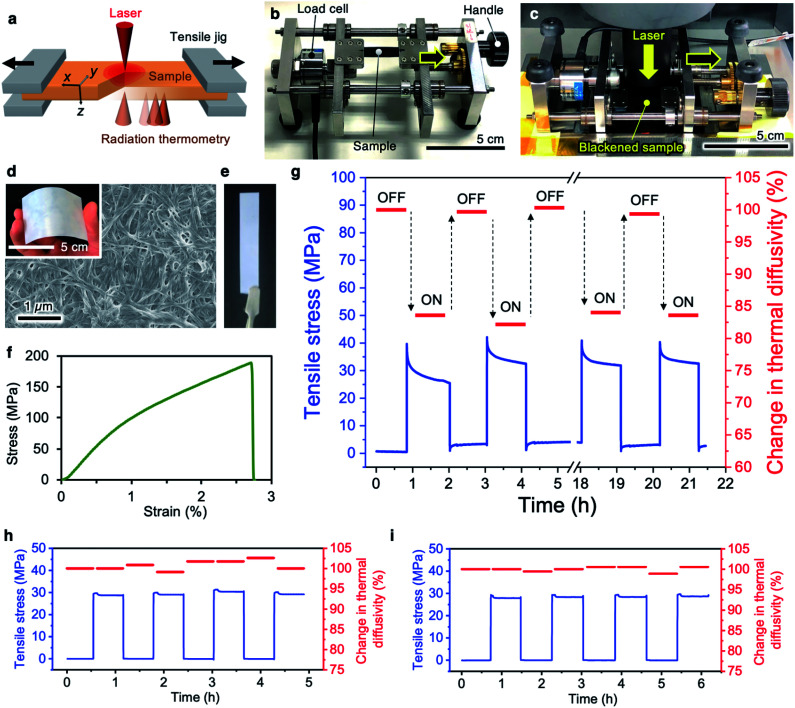
Thermal diffusivity modulation of CNF films. (a) Schematic illustration of measurement of the thermal diffusivity under an applied mechanical force. The sample was set on the jig (b) and introduced into the thermowave analyzer (c) without touching the jig chucks with the apparatus. (d) FESEM image of the bacterial CNF film surface. The inset shows the appearance of the flexible CNF film. (e) CNF film specimen (8 mm × 45 mm) used for the measurement. (f) Typical stress–strain curve of the CNF film. (g) Thermal diffusivity modulating behavior with repetitive stress loading–unloading. (h) and (i) Changes in the thermal diffusivities of copper and iron with mechanical stimulation, respectively.

The CNF films with two-dimensionally random fiber packing were produced by hot-pressing large-area *nata-de-coco* pellicles after purification. A field-emission scanning electron microscopy (FESEM) image of the CNF film surface is shown in Fig. 1d. An 8 mm × 45 mm specimen with thickness of 30–80 μm was used for the subsequent experiments (Fig. 1e). This film has high mechanical performance with Young's modulus of 12.4 GPa, 0.2% proof stress point of 109.1 MPa, and maximum strength of 189.3 MPa at strain of 2.7% (Fig. 1f). These values are comparable with the previously reported values for CNF sheets.^29,30,38^

By loading tensile stress of 30 MPa (with strain as small as ∼0.3%), the thermal diffusivity of the CNF film in the tensile (*x*) direction is about 15% lower than the initial value without stress, as shown in Fig. 1g. Through repetitive stress loading–unloading, switching behavior of the thermal diffusivity is observed for the CNF film. In contrast, the thermal diffusivities of copper and iron plates with no interior interfaces do not change (Fig. 1h and i, respectively). This agrees with the results for single silicon nanowires, which show no change in thermal conductivity with uniaxial strain.^8^ The sensitive switchability of the thermal diffusivity upon simple and small mechanical stimulation is therefore thought to be a characteristic property of CNF films.

The relative standard deviations for the thermal diffusivity measurements at the same point in a single CNF film were 2.345 and 1.402% in the in-plane and thickness directions, respectively (see Table S2 and Fig. S2†), and those at the five different points in a single CNF film were 1.020 and 5.439% (see Table S3 and Fig. S3†). The variation within the CNF film in the in-plane direction was at the same level with the measurement error of about 2%, and that in the thickness direction was a little larger, about 5%. On the other hand, the relative standard deviations in the measurement of ten different films in the in-plane and thickness directions were 16.27 and 25.91%, respectively. There is a large difference in diffusivity between different films due to the site differences in bacterial cellulose (BC) pellicles. Since the BC pellicles are formed by discharging CNF along with the movement of bacteria, there are large variations of fiber density and orientation.^39^ Even at applying ∼34 MPa, the relative standard deviations for the thermal diffusivity of the CNF film were found as small as 1.67, 2.64 and 3.95% in *x*, *y*, and *z* directions, respectively.

To clarify the directional dependence of the thermal diffusivity change with the external force, the thermal diffusivities in the *x*, *y*, and *z* directions were separately measured for the CNF film by applying stepwise stresses, as shown in Fig. 2a. The thermal diffusivities in the *x* and *y* directions are 1.4–1.8 mm^2^ s^−1^ and that in the *z* direction is only 0.4–0.5 mm^2^ s^−1^ (Fig. 2b), which reflects the fiber alignment within the films of two-dimensionally random orientation in the in-plane direction and layer stacking in the thickness direction.^26^ To highlight the diffusivity change, the change rates are plotted against the average stresses in the *x*, *y*, and *z* directions for four different specimens in Fig. 2c. Only the thermal diffusivity in the *x* direction clearly decreases with increasing stress (by almost 15%), whereas those in the *y* and *z* directions remain constant considering the measurement error range of the thermal diffusivity of ±5% owing to the measurement accuracy and location variation of the CNF films. Considering the large variations in the absolute thermal diffusivity between the different CNF films, the diffusivity responses in *x* direction to the applied stress were well converged and considered a reliable change. In addition, the diffusivity reduction in the *x* direction decreases with increasing applied stress. We consider that these two types of diffusivity behavior reflect the switching mechanism: (1) diffusivity reduction in the *x* direction and (2) leveling off of the diffusivity reduction at high stress.

**Fig. 2:**
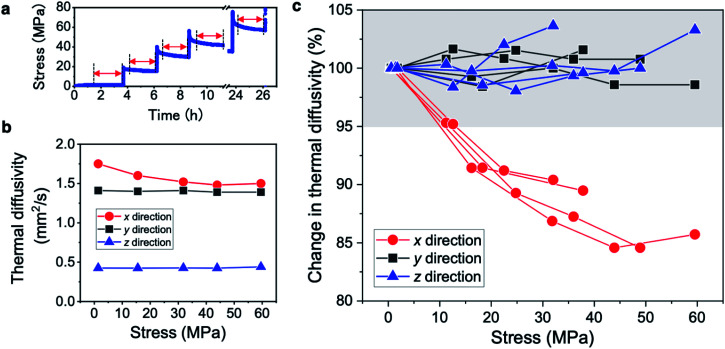
Thermal diffusivity modulation behavior of the CNF film in the *x*, *y*, and *z* directions. (a) Applied stepwise stress profile. (b) Thermal diffusivities in the *x*, *y*, and *z* directions corresponding to those defined in Fig. 1a. (c) Relationship between the average stress and thermal diffusivity in each direction for four specimens. The gray shaded region indicates the measurement error range of the thermal diffusivity of ±5%.

To reveal the mechanical deformation mode, we analyzed the stress relaxation profiles of CNF films at different stress levels (Fig. 3a). Viscoelastic relaxation is expressed by the Maxwell model with an elastic spring and a viscous damper connected in series. We first fitted the experimental profile with the Maxwell equation with one relaxation time *τ* (eqn (1) in the Method section). The coefficient of determination is 0.987 with a poorly fitted profile (Fig. 3b). This indicates that the relaxation profiles have multiple superimposed relaxation times rather than a single relaxation time. We then fitted the generalized Maxwell models^40,41^ with two and three *τ* parameters (eqn (2) and (3) in the Method section) to the profiles. The highest coefficient of determination of 0.999 is obtained with the three-parameter model (right part of Fig. 3b). When the models with four and five *τ* parameters were used, the fitted results only showed three *τ* values, so the CNF films have three superimposed relaxation times.

**Fig. 3:**
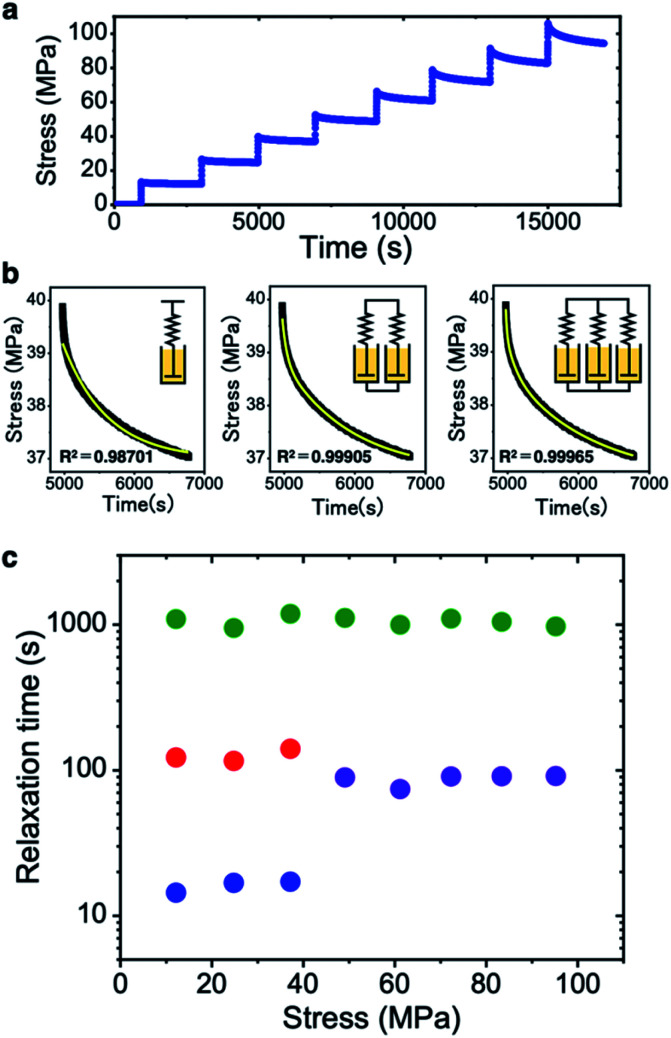
Relaxation mode analysis of the CNF film. (a) Stress relaxation profile for stepwise stress loading. (b) Each relaxation profile fitted by generalized Maxwell models with different numbers of relaxation time parameters. (c) Relationship between the average stress and relaxation times for the CNF film.

The *τ* parameters are plotted against the stress level in Fig. 3c. For the stress levels below ∼40 MPa, there are three *τ* values on the order of 10, 100, and 1000 s, which are thought to correspond to extensible elastic deformation of hydrogen bonds, slipping between CNFs, and plastic deformation of the whole network, respectively. This indicates that the elastic deformation mode is dominant for the reversible responses of both the elasticity^31,32^ and thermal diffusivity (Fig. 1g). Above 40 MPa stress, the three *τ* values converge to two *τ* values on the order of ∼100 and 1000 s (*i.e.*, there is no *τ* value with 10 s order). This change is thought to reflect the yield point where the relaxation mode shifts to the plastic-deformation-dominated mode. The hydrogen bonding is stressed beyond the elastic limit and slipping deformation becomes dominant, because the hydrogen bonds on the CNF surface can reform after fracturing.^33^ The two *τ* values are maintained at high stress until the film breaks.

The two types of thermal diffusivity change behavior, (*i.e.*, (1) diffusivity reduction in the *x* direction and (2) leveling off of the diffusivity reduction at high stress, see Fig. 2c) can be explained from the relaxation time behavior (Fig. 3c). Regarding (1), low stress allows the CNF film to elastically deform at the CNF interfaces to reversibly change the interfacial thermal resistance, and responsive or linear reduction of the diffusivity to small stress levels is observed. Regarding (2), high stress makes the film shift to the plastic deformation mode and the interfacial responsiveness is impaired, leading to slower diffusivity reduction.

The bulk parts of the CNFs also show strain to applied stress,^38^ and they are thought to affect phonon conduction, as previously predicted for various materials through simulations.^14–22^ CNFs have positive Poisson's ratios of ∼0.37 to 0.64 depending on the crystallographic planes against tension in the axial direction,^42^ and the CNFs in the films can slightly slenderize by uniaxial strain. It has also been reported that the CNF thickness is linearly related to the thermal diffusivity of the film owing to the crystallite size effect,^26^ so CNF slenderization by the Poisson effect with applied stress could in principle occur to decrease the thermal diffusivity. However, according to the reported relationship,^26^ the cross-sectional area of the CNFs would need to decrease by ∼20% to reduce the thermal diffusivity of the film by 10–15%. It is unrealistic to reduce the cross-sectional area of CNFs with Poisson's ratios of ∼0.37 to 0.64 by 20% because of the very small strain of ∼0.3% at 30 MPa stress. In addition, our films with two-dimensionally random orientation of CNFs are unable to exert uniaxial strain to unidirectionally deform each CNF. That is, the CNFs in the films show little deformation under ∼30 MPa stress, and the small total strain of ∼0.3% converges on the CNF interfaces to slightly change the bonding distances within the range of elastic deformation. The deformation in the *x* direction of CNF film was thought caused by the interfacial strains contributing to the adhesive force in the *x* direction. Taking into account that clear directional dependence of thermal diffusivity modulation is observed in Fig. 2c despite non-unidirectional deformation of the CNFs occurring in the films, we conclude that the large thermal diffusivity changes of the CNF films can only be explained by the interfacial dynamics between the CNFs.

To further demonstrate the effect of “interfacial dynamics” on the thermal diffusivity switching by changing the interfacial bonding area of CNFs, we prepared the CNF films with greatly different bulk densities from 0.75 to 1.33 g cm^−3^, which were thought the lower and upper limit densities for BC-derived CNF films to test the mechanical modulation, and tested the thermal diffusivity change under the ∼30 MPa stress. The average changes in thermal diffusivity were defined to be the differences between the respective average diffusivities at the four times of loading or unloading as the “on/off loading” samples, while the available data at the stress of ∼30 MPa were picked up from Fig. 2c as the “stepwise loading” samples. As shown in Fig. 4a and S6,† the thermal diffusivity modulation rate greatly varied in proportion to the bulk density. In addition, the original thermal diffusivity for each film before loading varied inversely to the bulk density (Fig. S6f†). The mechanical modulation of thermal diffusivity on the CNF films were demonstrated driven by the interfacial dynamics between CNFs, closely related with the bulk density.

**Fig. 4:**
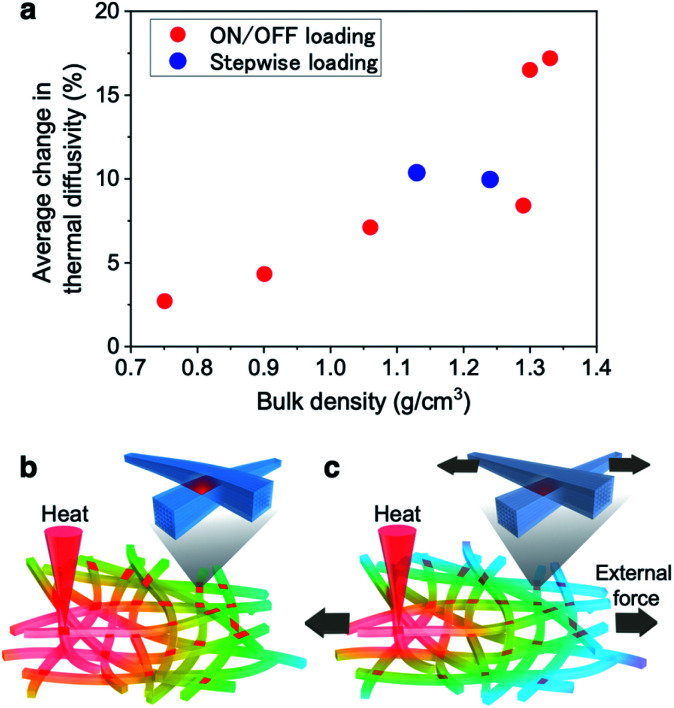
Interfacial elastic dynamics between CNFs affecting thermal transport modulation. (a) The relationship between the bulk density of CNF films reflecting the CNF interfacial density and the thermal diffusivity change. (b) The schematic illustration showing that the CNFs in the film without any stress strongly joined by hydrogen bonding to smoothly transfer heat. (c) Application of an external force to the CNF film, which increases the interfacial thermal resistance at the partial CNF interfaces to inhibit heat conduction.

The thermal diffusivity switching mechanism depending on the CNF-interfacial dynamics is schematically illustrated in Fig. 4b and c. The CNF bonding planes in the film face in various directions because of the two-dimensionally random orientation (Fig. 1d). The partial faces among the bonding planes, whose directions are thought to be approximately parallel to the loading directions,^33^ bear the load to conduct the stresses from end to end with large distributions of local strain.^43^ The bonding planes in other directions may not dominantly deform to show clear directional dependence, as shown in Fig. 2c.

To further demonstrate thermal diffusivity modulation by the elastic dynamics of the CNF interfaces, we performed a proof-of-concept experiment by designing a continuous stress profile, as shown in Fig. 5. The thermal diffusivity responsively changes in the early period of applying repetitive stress of 0 to 40 MPa, exhibiting a thermal diffusivity change from 1.58 to ∼1.45 mm^2^ s^−1^ owing to the elastic deformation mode of the CNF film, similar to Fig. 1g. Subsequently, by applying higher stress of ∼60 MPa on average from 21 to 33 h, the diffusivity continuously measured fifteen times does not change throughout this period and remains lower than that at 0 MPa stress. In addition, the diffusivities of ∼1.44 mm^2^ s^−1^ under 60 MPa are almost the same as those of ∼1.45 mm^2^ s^−1^ at applied stress of 40 MPa during the early period. The progression of plastic deformation at higher stress makes the elastic response weaker to slow the diffusivity reduction, corresponding to the results shown in Fig. 2c. After unloading the 60 MPa stress and repeating loading and unloading with 40 MPa, the diffusivity change becomes weak and irregular during the period from 40 to 46 h. This behavior was also confirmed from the fact that the changes in thermal diffusivity were found to be 5.93 ± 1.26%, −5.26 ± 1.24%, and 30.0 ± 16.5% at the excessive stress of ∼91 MPa in *x*, *y*, and *z* directions, respectively. These thermal diffusivity modulation behaviors correspond well with the concept of the interfacial elastic dynamics between the CNFs, as shown in Fig. 4.

**Fig. 5:**
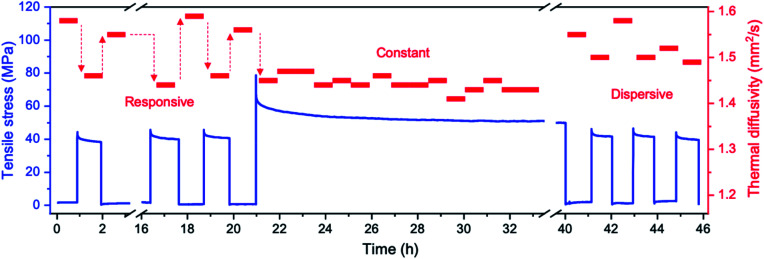
Proof-of-concept demonstration by a continuous experiment to demonstrate the thermal diffusivity modulator driven by the interfacial elasticity between the CNFs.

## Conclusions

In conclusion, we have succeeded in realizing a thermal diffusivity modulator based on a mechanically tough CNF film by simply applying a very small mechanical strain through developing the combinational measuring system for the bulk material. The interfacial elastic dynamics between the strongly hydrogen-bonded CNFs exhibit thermal diffusivity changes by tuning the interfacial thermal resistance against the strain, rather than changing the bulk structure of the CNFs. Because interfacial resistance for thermal transport can be tuned based on the chemically interacting groups,^44^ grain boundary conditions,^45^ and interface density owing to the anisotropic (rod-like,^36^ fibrous,^25^ or placoid^46^) element orientations, the modulation rate or sensitivity of thermal conduction could possibly be enhanced by designing the interfacial conditions. We envision that our concept of interface-driven thermal conduction modulation could be extended toward practical use as the key thermal technique to address general heat issues.

## Methods

Bacterial cellulose pellicles (*i.e.*, *nata de coco* sheets) produced by incubating *Acetobacter xylinum* were purchased from Fujicco Co., Ltd. (Kobe, Japan). The 10–15 mm thick pellicles were boiled in 2 wt% NaOH aqueous solution at 80–90 °C for 2 h, followed by thorough washing with distilled water for 5 days to remove the bacterial cell debris. After purification, the pellicles were hot-pressed at 115 °C for 20 min to obtain pristine CNF films with 30–80 μm thickness and bulk density of 1.1–1.2 g cm^−3^. The moisture content under the relative humidity of 10–15% was ∼2.98 wt% estimated through the thermogravimetry by a Q50, TA Instruments (USA).

The additional CNF films with high bulk densities exceeding 1.3 g cm^−3^ with ∼40 μm thickness were prepared as follows: a CNF film dried by hot-pressing described above was immersed in distilled water for 5 min and sandwiched between metal meshes (300 mesh), followed by additional hot-pressing at 110 °C for 5 min. The cycle of water immersion and hot-pressing were repeated 5 times.

The additional CNF films with low bulk densities below 1.1 g cm^−3^ were made as follows: the purified BC pellicle was compressed about 10 times without applying heat to form a wet compressed hydrogel with 50 × 50 × 1 mm size. The hydrogel was immersed in *tert*-butyl alcohol for 2 hours and again immersed in a new *tert*-butyl alcohol for 24 hours to replace the water to alcohol. The alcogel was then sandwiched between metal meshes (300 mesh) and hot-pressed at 110 °C with a small load with 0.1 MPa or less to pre-dry for 2 to 4 min. The pre-drying time determined the final bulk density: a 2 min pre-dried gel resulted in a low-density film of 0.75 g cm^−3^ with ∼80 μm thickness, and the 3 min and 4 min pre-dried gels for 0.90 (∼90 μm thickness) and 1.06 g cm^−3^ (∼65 μm thickness), respectively. After preliminary drying, the gels still containing alcohol were froze in liquid nitrogen and freeze-dried overnight to obtain the low bulk-density dry films.

FESEM (SU8020, Hitachi High-Tech. Corp., Tokyo, Japan) was performed to image the film surfaces at an accelerating voltage of 2 kV with a Pt coating (*ca.* 1 nm) applied by ion sputtering. The stress–strain curves of the CNF films were recorded with an EZ-SX universal testing machine (Shimadzu Corp., Kyoto, Japan) with the cross-head speed of 10 mm min^−1^.

The samples were blackened on both sides by FC-142 spray (Fine Chemical Japan Co., Ltd., Tokyo, Japan) to make them absorb the laser energy on the surface and maintain high emissivity for detecting the temperature wave by the radiation thermography on the back side.^26,28^ After spraying, the sample was held over light to check for impermeability, and the surface was rubbed with a paper towel to remove excess graphite, and reduce the blackened layer thickness for both sides with a total ∼15 μm or less. The blackened films had neither spray spots nor large unevenness thin and uniform. A specially designed tensile jig (TJ-161, Bethel Co., Ltd., Ibaraki, Japan) with a 200 N load cell was used to perform the thermal diffusivity measurements through a TA33 thermowave analyzer (Bethel, Co., Ltd.), which complies with the laser spot periodic heating radiation thermometry method.^28^ The handle of the jig was manually rotated to apply tensile strain to the held film sample with 25 mm between chucks, and the strain remained constant after releasing handle. After waiting for ∼15 min for stress relaxation to subside, the thermal diffusivity measurement was started at a particular point of the sample to avoid detecting the regional differences. The measurements were performed under the room temperature strictly kept at 25 ± 2 °C at relative humidity of 10–40% in order to maintain the accuracy of the radiation thermometer, and the chamber door part of the TA33 was covered with a black curtain to prevent air current disturbance and detection of external light.

Nonlinear fitting of the stress relaxation curves was performed with Origin Pro version 2019 (OriginLab Corp., Northampton, MA, USA) using the generalized Maxwell models of the exponential decay functions with one (eqn (1)), two (eqn (2)) and three (eqn (3)) time constant parameters *τ* with the Levenberg–Marquardt iteration algorithm:1
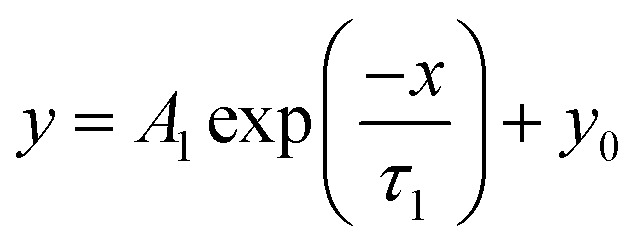
2
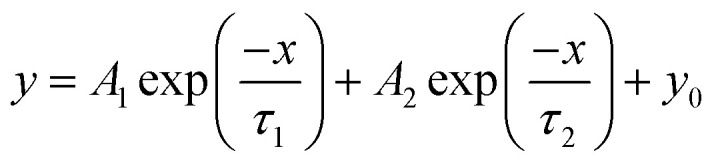
3
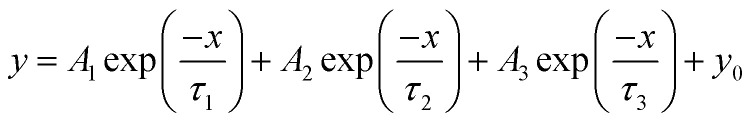
where *y*_0_, *A*, and *τ* are the offset, amplitude, and time constant (relaxation time), respectively.

## Conflicts of interest

The authors declare no competing financial interest.

## Supplementary Material

NA-002-C9NA00734B-s001
