# Supplementary material for: Thermal diffusivity modulation driven by the interfacial elastic dynamics between cellulose nanofibers
Source: Nanoscale Adv. 2020 Jan 20;2(3):1024–30. doi: 10.1039/c9na00734b (PMC9417970; doi:10.1039/c9na00734b)
Supplement: NA-002-C9NA00734B-s001 [file NA-002-C9NA00734B-s001.pdf]

Electronic Supplementary Material (ESI) for Nanoscale Advances.
This journal is © The Royal Society of Chemistry 2020

Supporting information

Thermal Diffusivity Modulation Driven by the Interfacial Elastic Dynamics Between Cellulose Nanofibers

Kojiro Uetani[1,*], Shogo Izakura[2], Hirotaka Koga[1], Masaya Nogi[1]

[1]The Institute of Scientific and Industrial Research (ISIR), Osaka University, Mihogaoka 8-1, Ibaraki-shi, Osaka 567-0047, Japan

[2]Graduate School of Engineering, Osaka University, Mihogaoka 8-1, Ibaraki-shi, Osaka 567-0047, Japan

This PDF file includes:

Figure S1 to S6
Table S1 to S3

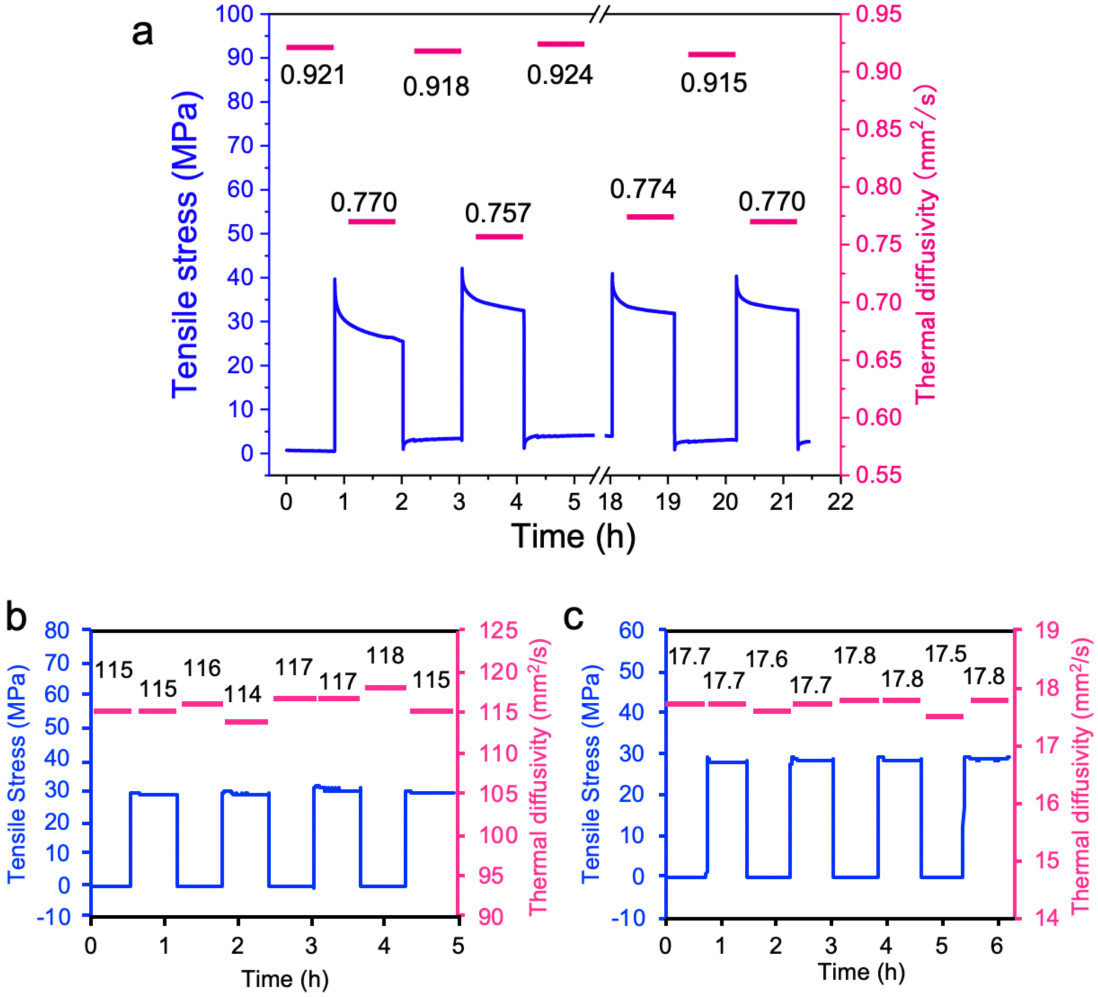

Figure S1. The measured thermal diffusivity values for the CNF film (a), cupper (b) and (c) iron plates, shown in Figure 1.

Table S1. Variability evaluation of repetitive measuring thermal diffusivity at the same point in a single CNF film.

	In-plane thermal diffusivity (mm^2/s)	Through-plane thermal diffusivity (mm^2/s)
No.1	1.26	0.226
No.2	1.20	0.227
No.3	1.24	0.219
No.4	1.18	0.221
No.5	1.21	0.221
Average	1.218	0.2228
SD	0.029	0.0031
Relative SD (%)	2.345	1.402

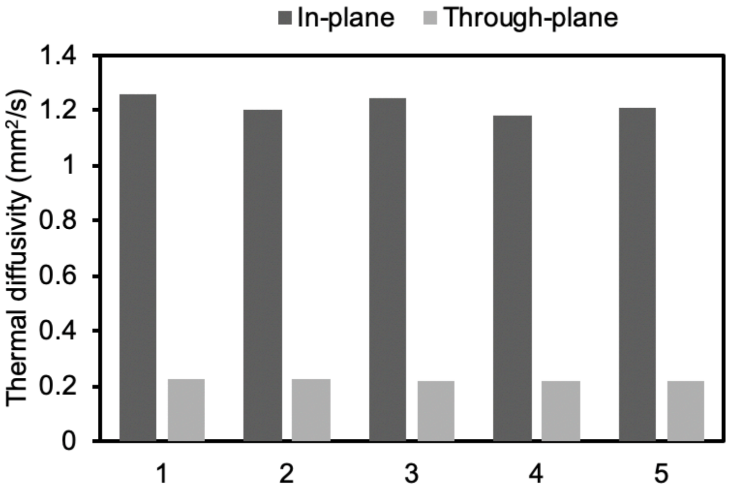

Figure S2. Visualization of Table S1.

Table S2. Variability evaluation of measuring thermal diffusivity at the 5 different points within a single CNF film.

	In-plane thermal diffusivity (mm^2/s)	Through-plane thermal diffusivity (mm^2/s)
No.1	1.17	0.17
No.2	1.17	0.144
No.3	1.16	0.157
No.4	1.19	0.153
No.5	1.19	0.153
Average	1.176	0.1554
SD	0.012	0.0085
Relative SD (%)	1.020	5.439

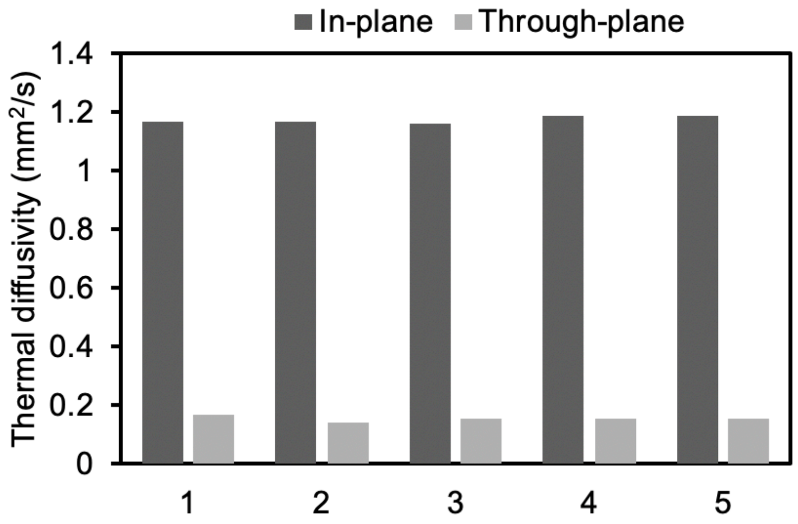

Figure S3. Visualization of Table S2.

Table S3. Variability evaluation of measuring thermal diffusivity on the 10 different CNF films.

	In-plane thermal diffusivity (mm^2/s)	Through-plane thermal diffusivity (mm^2/s)
No.1	1.01	0.205
No.2	0.909	0.179
No.3	0.805	0.172
No.4	0.926	0.131
No.5	0.895	0.115
No.6	1.25	0.25
No.7	1.11	0.243
No.8	1.18	0.183
No.9	1.35	0.144
No.10	1.23	0.123
Average	1.067	0.175
SD	0.174	0.0452
Relative SD (%)	16.270	25.906

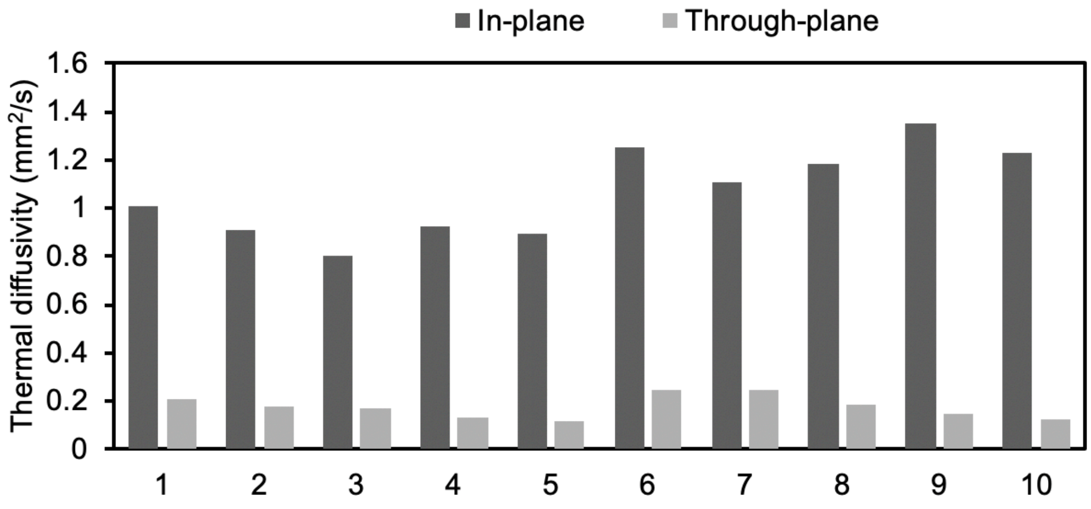

Figure S4. Visualization of Table S3.

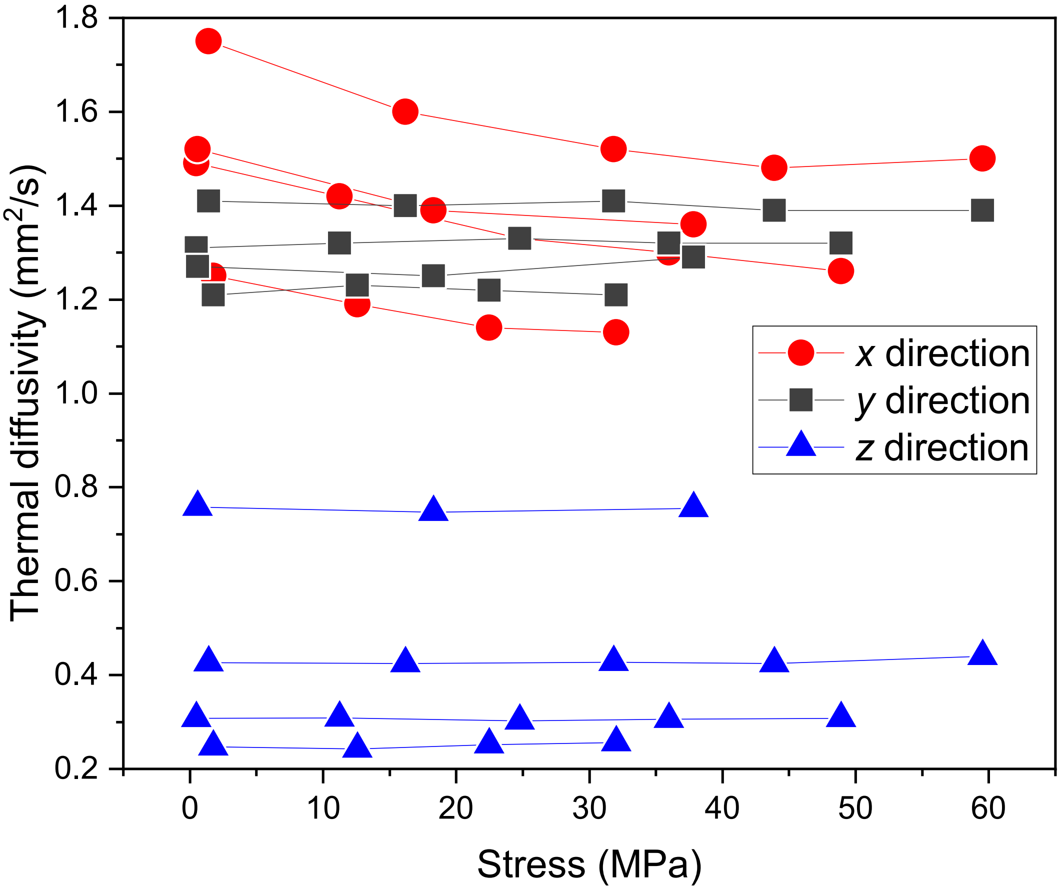

Figure S5. The measured thermal diffusivities for four CNF films shown in Figure 2c.

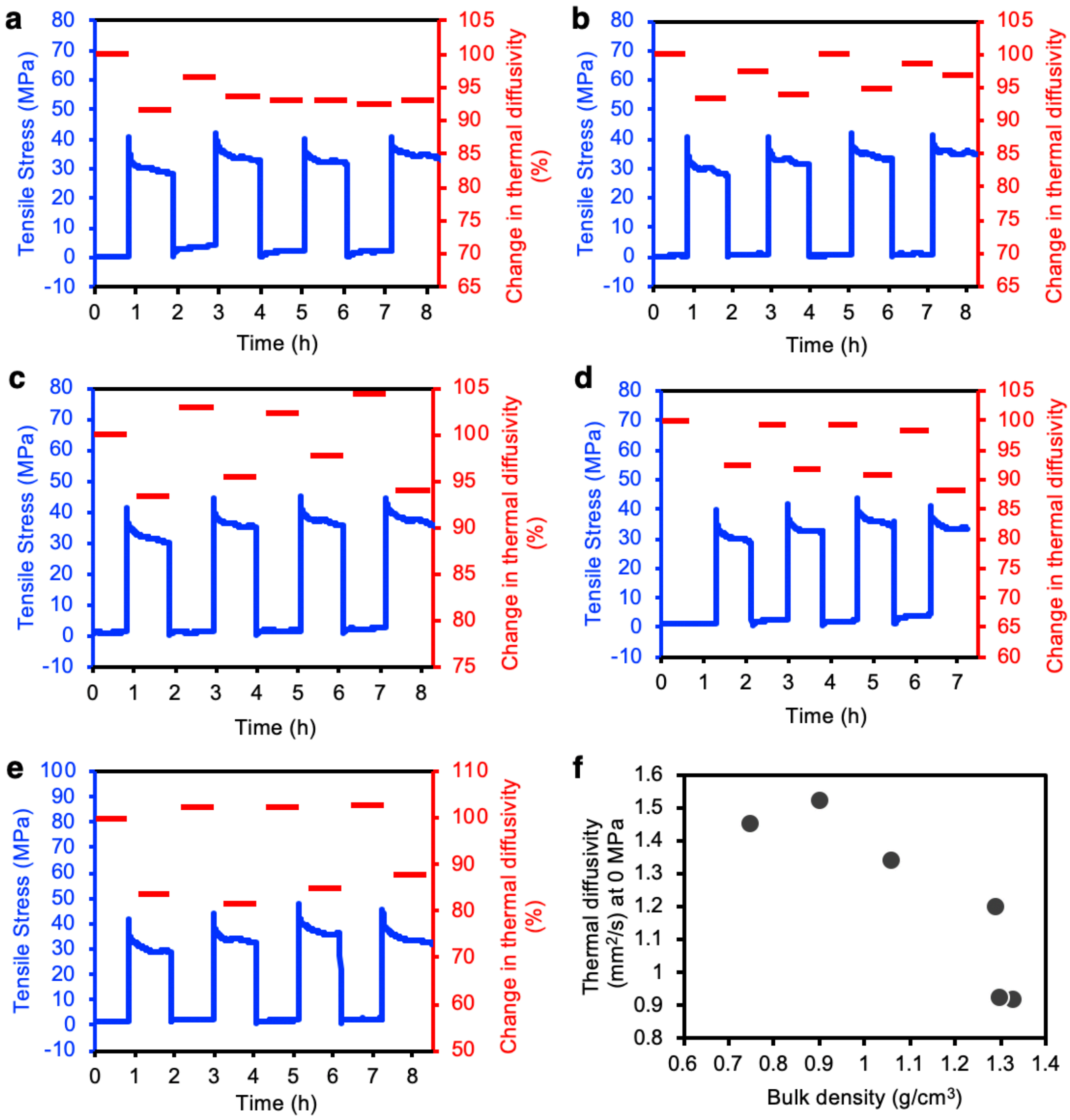

Figure S6. Thermal diffusivity modulation behaviors for CNF films with different bulk density of (a) 0.751, (b) 0.901, (c) 1.06, (d) 1.29, and (e) 1.33 g/cm^3. (f) The original thermal diffusivity for each films before applying stress varied inversely to the bulk density.
